# Comparative Effects of Cigarette and Hookah Smoking on Oral Microbiota Composition and Blood Indices in Young Adult Males

**DOI:** 10.1096/fba.2025-00296

**Published:** 2026-06-08

**Authors:** Fayez Alghofaili, Hemin Mohamad Hussein, Hastyar Hama Rashid Najmuldeen, Soma Yasin Tahsin, Asia Hamasaeed Muhamad, Ara Jabbar Othman, Kashan Faraidun Karim, Aryan Omer Ali, Ala Salam Karim

**Affiliations:** ^1^ Department of Medical Laboratory Sciences Majmaah University Majmaah Saudi Arabia; ^2^ Department of Biology, College of Science University of Sulaimani Sulaymaniyah Iraq; ^3^ College of Health Sciences Cihan University Sulaimaniya Sulaymaniyah Iraq; ^4^ Department of Medical Laboratory Science Komar University of Science and Technology Sulaymaniyah Iraq

**Keywords:** cigarette smoking, correlation analysis, hematological parameters, hookah smoking, oral microbiota

## Abstract

Cigarette and hookah smoking remain prevalent worldwide, yet their comparative effects on oral microbiota and hematological parameters are not fully understood. This cross‐sectional study investigated these impacts among 87 healthy males (18–40 years) in Sulaymaniyah, Iraq, divided into cigarette smokers, hookah smokers, and non‐smokers (*n* = 29 each). Oral rinses were analyzed for microbial load and species distribution using selective media and standard identification techniques, while venous blood samples were evaluated for complete blood counts using an automated analyzer. Cigarette smokers exhibited the highest oral bacterial load (5.96 ± 0.19 log_10_ CFU/mL) compared with non‐smokers (3.70 ± 0.10; *p* < 0.001), followed by hookah smokers. Colonization by *Candida albicans* and Gram‐positive cocci (Staphylococcaceae, Streptococcaceae) was more frequent in smokers. Both smoking methods significantly increased RBC count, hemoglobin, and hematocrit levels (*p* < 0.01). Mean corpuscular volume increased significantly only in cigarette smokers, whereas red cell distribution width and mean platelet volume were significantly higher in hookah smokers (*p* < 0.01). Exploratory correlation analysis revealed strong positive associations between oral bacterial load and erythrocyte‐related parameters (RBC, HGB, HCT; *r* = 0.91–0.94), while weak correlations were observed with inflammatory and platelet indices (WBC, RDW, MPV). These findings indicate that cigarette and hookah smoking disrupt oral microbial balance and alter hematological parameters, with distinct patterns between smoking types. The observed correlations likely reflect parallel systemic effects of smoking‐related toxicants rather than a direct causal link between oral dysbiosis and erythropoiesis. Overall, the results reinforce that hookah smoking is not a safer alternative to cigarette use and highlight the need for targeted public health interventions.

## Graphical abstract

This study examined 87 healthy young males to compare the effects of cigarette and hookah smoking on oral microbiota and hematological parameters. Cigarette smokers showed the highest oral bacterial load and greater colonization by *Candida albicans* and Gram‐positive cocci, while hookah smokers also exhibited elevated microbial levels. Both smoking types significantly increased RBC, hemoglobin, and hematocrit compared with non‐smokers, with hookah smoking producing stronger hematological effects.

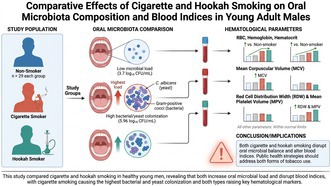

## 1 Introduction

Tobacco use remains a critical global health challenge, with 1.3 billion users worldwide and over eight million deaths annually, including 1.6 million attributed to second‐hand smoke [1]. Among Arab countries, cigarette and hookah (waterpipe) smoking are highly prevalent, particularly among young males, with rates reaching 46.7% in Egypt and 46% in Kuwait [2]. Despite widespread misconceptions, hookah smoking is not a safer alternative; it exposes users to toxicants including nicotine, carbon monoxide, oxidants, and free radicals, all of which promote oxidative stress and systemic disease [3, 4]. A single hookah session delivers nicotine levels comparable to two or three cigarettes, alongside harmful particulate matter that induces inflammation and oxidative damage [5, 6, 7]. Epidemiological evidence links hookah smoking to cardiovascular complications and a nearly twofold increase in ischemic heart disease mortality [5].

Smoking's adverse health effects extend beyond systemic risks to oral health, where it alters the composition of the oral microbiota. This complex ecosystem, essential for homeostasis, can become dysbiotic under smoking‐induced stress, favoring opportunistic pathogens implicated in periodontal disease and systemic inflammation [8]. Cigarette smoking enriches pathogenic species such as *Fusobacterium*, *Prevotella*, *Porphyromonas*, and *Tannerella*, while depleting non‐pathogenic microbiota [9, 10, 11]. Similar disruptions have been observed with hookah smoking, which significantly alters microbial diversity and biochemical profiles, increasing infection risk, including *Helicobacter pylori* colonization [12, 13].

In addition, smoking impacts hematological parameters through mechanisms such as tissue hypoxia caused by carbon monoxide binding to hemoglobin, leading to compensatory erythrocytosis and increased hematocrit [14, 15]. Although previous studies have examined these effects in cigarette smokers, findings remain inconsistent, and comparative data on hookah and cigarette smokers are scarce [16, 17]. Understanding these hematological changes is essential, as they may signal early cardiovascular risk or polycythemia development [18, 19].

Recent evidence from Sudan [20] shows that cigarette and hookah smokers exhibit significant effects on some hematological parameters compared with non‐smokers. These consistent patterns indicate that smoking‐related hematological changes are reproducible across populations. To provide region‐specific evidence, the present study integrates oral microbiological profiling with hematological assessment by comparing oral microbial composition and complete blood count parameters among cigarette smokers, hookah smokers, and non‐smokers in Sulaymaniyah City, Iraq. This dual approach offers insights into both local and systemic effects of tobacco use, contributing to the evidence base for targeted public health interventions.

## 2 Methodology

### 2.1 Study Design and Participants

This cross‐sectional study was conducted in Sulaymaniyah, Iraq, from December 2018 to March 2019. A total of 87 healthy males aged 18–40 years were enrolled and divided into three equal groups: cigarette smokers, hookah smokers, and non‐smokers (*n* = 29 each). Cigarette smokers had smoked at least 100 cigarettes in their lifetime, while hookah smokers reported a minimum of 1 year of use. Exclusion criteria included chronic diseases (diabetes, cardiovascular, renal, or endocrine disorders), recent surgery, medication use (β‐blockers, steroids, lipid‐lowering agents), alcohol consumption, and recent antibiotic use. Females were excluded due to cultural factors limiting smoking prevalence. Informed consent was obtained from all participants. Ethical approval was granted by the Ethics Committee of Cihan University Sulaimaniya (Reference No. HS‐1‐2018).

### 2.2 Microbiological Analysis

#### 2.2.1 Sample Collection

Participants rinsed with 10 mL sterile saline for 30 s and expectorated into sterile containers, following Ahmed et al. [21]. Samples were transported in cool boxes for immediate processing.

#### 2.2.2 Microbial Load

The viable plate count method was employed using tenfold serial dilutions. Twenty‐five microliters from appropriate dilutions were inoculated on nutrient agar, incubated at 37°C for 18 h, and colony‐forming units (CFU/mL) calculated [22].

#### 2.2.3 Microbial Isolation and Identification

Culture‐dependent methods were used to characterize the cultivable microbiota. Selective and differential media included MacConkey agar for Enterobacteriaceae, Mannitol salt agar for *Staphylococcaceae*, Blood agar for *Streptococcaceae*, and Sabouraud dextrose agar for fungal isolation. MacConkey and Mannitol salt agar plates were incubated aerobically at 37°C for 24 h. Blood agar plates were incubated at 37°C for 24 h under microaerophilic conditions using the candle jar technique to support optimal growth of *Streptococcaceae*. Sabouraud dextrose agar plates were incubated at 25°C for 72 h.

Presumptive identification was based on colony morphology and Gram staining. Gram‐negative rods were recorded for presumed Enterobacteriaceae, Gram‐positive cocci in clusters for *Staphylococcus* spp., Gram‐positive cocci arranged in chains for *Streptococcus* spp., and fungal elements were identified microscopically as yeast cells or filamentous hyphae. Biochemical confirmation included catalase, coagulase, and oxidase tests following standard protocols ([23]; Bergey's Manual of Systematic Bacteriology [24]).

### 2.3 Hematological Analysis

Venous blood samples (5 mL) were collected in EDTA tubes and analyzed within 3 h using a fully automated hematology analyzer (HA‐22 CLINDIAG). Complete blood counts included RBC, HGB, HCT, MCV, MCH, MCHC, platelets, MPV, WBC, and differential counts. Normal reference values were based on Adeli et al. [25].

### 2.4 Statistical Analysis

Data were analyzed using GraphPad Prism 9. Results are presented as mean ± standard deviation (SD). Group comparisons were performed using one‐way ANOVA followed by Dunnett's post hoc test, with statistical significance set at *p* < 0.05, *p* < 0.01, and *p* < 0.001.

In addition, an exploratory correlation analysis was conducted to assess the relationship between oral microbial load (Log_10_ CFU/mL) and hematological parameters (RBC, HGB, HCT, WBC, RDW, and MPV). Due to the absence of individual‐level paired data, Pearson correlation coefficients (*r*) were calculated using group‐level mean values. The results were interpreted descriptively to identify trends rather than to infer causality.

## 3 Results

### 3.1 Effect of Smoking on Oral Microbiota

**FIGURE 1:**
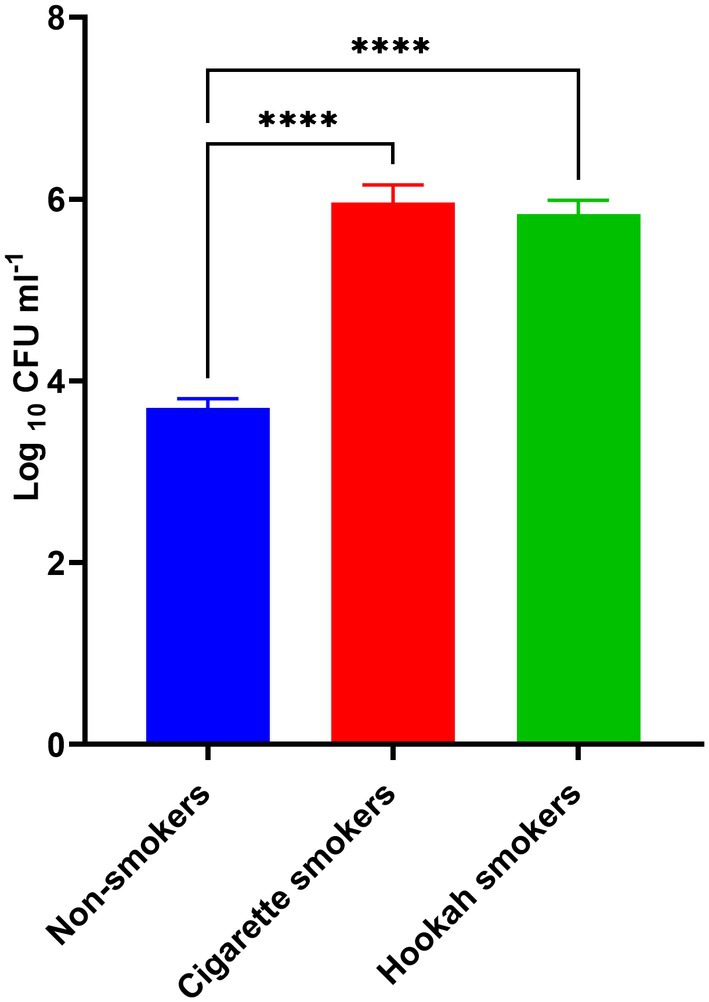
Effect of cigarette and hookah smoking on oral bacterial load. Bacterial counts were determined using the viable plate counting method and expressed as Log_10_ CFU/mL. Data are shown as mean ± S.D. Statistical differences were assessed using one‐way ANOVA with Duncan's multiple comparisons test. ****p < 0.0001 indicates highly significant differences compared with the non‐smoker control group.

The viable plate count analysis revealed a significantly higher oral bacterial load in cigarette smokers (5.961 ± 0.19 Log_10_ CFU/mL) compared to non‐smokers (3.702 ± 0.10 Log_10_ CFU/mL; *p* ≤ 0.001). Hookah smokers also demonstrated increased microbial load (2.834 ± 0.15 Log_10_ CFU/mL) relative to controls (*p* ≤ 0.001) (Figure 1).

**FIGURE 2:**
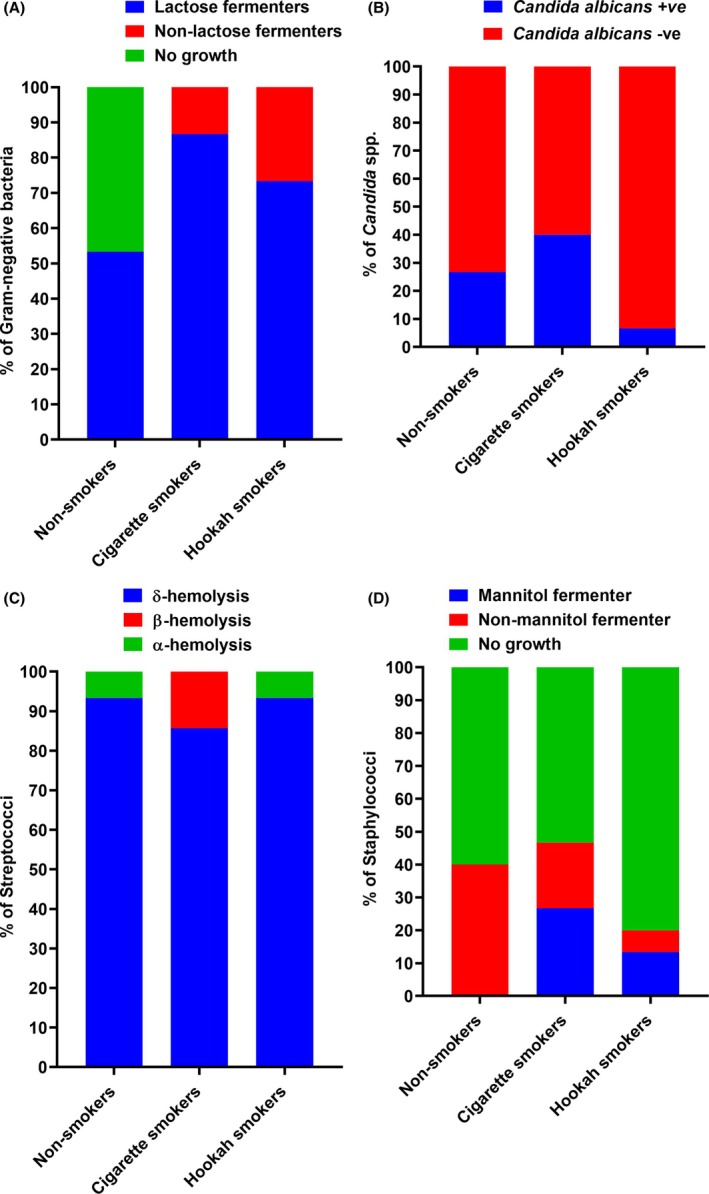
Distribution of oral microorganisms among study groups (A) Gram‐negative lactose and non‐lactose fermenters isolated on MacConkey agar. (B) *Candida albicans* isolated on Sabouraud dextrose agar. (C) Hemolytic patterns of oral *streptococci* on blood agar, showing proportions of α‐, β‐, and γ‐hemolysis. (D) *Staphylococcus aureus* (mannitol fermenters) and non‐mannitol fermenters isolated on Mannitol salt agar. Percentages indicate the proportion of positive samples within cigarette smokers, hookah smokers, and non‐smokers.

MacConkey agar results indicated that lactose‐fermenting bacteria were more prevalent than non‐lactose fermenters across all groups. Cigarette smokers had the highest proportion of lactose fermenters (86.67%), followed by hookah smokers (73.33%) and non‐smokers (53.33%). In contrast, non‐lactose fermenters were more frequent in hookah smokers (26.67%) and cigarette smokers (13.33%), whereas non‐smokers showed no growth on MacConkey agar (46.67% negative growth) (Figure 2A). *Candida albicans* prevalence was greatest in cigarette smokers (40%), compared to non‐smokers (26.67%) and hookah smokers (6.67%) (Figure 2B).

Blood agar cultures showed that γ‐hemolysis predominated across groups, with the highest proportions in non‐smokers (93.33%) and hookah smokers (93.33%), followed by cigarette smokers (85.71%). β‐hemolysis was observed exclusively in cigarette smokers (14.29%), while α‐hemolysis appeared in non‐smokers and hookah smokers (6.67%) but not in cigarette smokers (Figure 2C).

On Mannitol salt agar, all mannitol‐fermenting *staphylococci* were confirmed as *Staphylococcus aureus* . Cigarette smokers exhibited the highest prevalence of *S. aureus* (28.57%), followed by hookah smokers (14.29%); none was detected among non‐smokers (Figure 2D). Non‐mannitol fermenters were most frequent in non‐smokers (40%), compared to cigarette (21.43%) and hookah smokers (6.67%).

### 3.2 Effect of Smoking on Hematological Parameters

**FIGURE 3:**
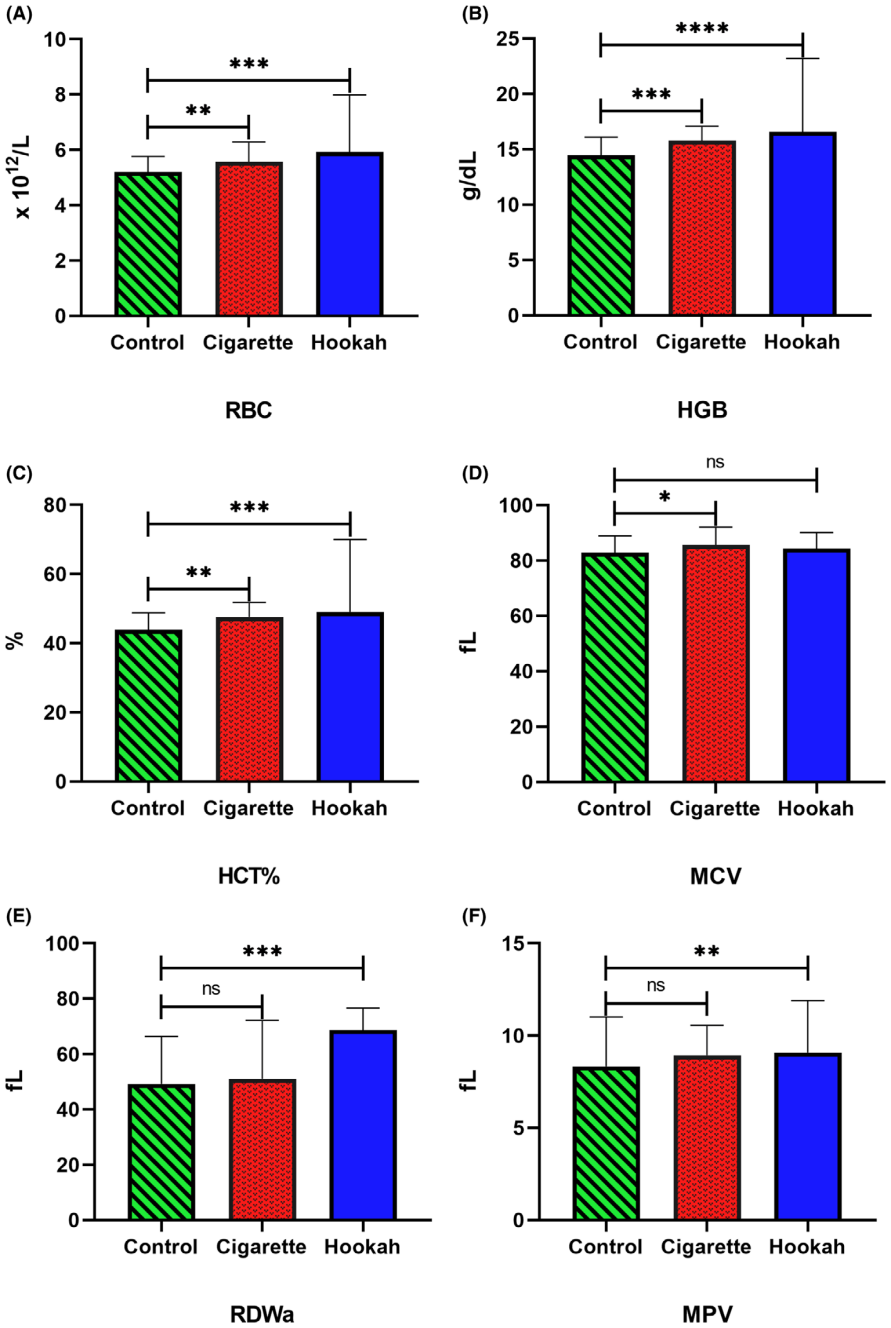
Effect of cigarette and hookah smoking on hematological parameters. Comparison of (A) red blood cell count (RBC), (B) hemoglobin concentration (HGB), (C) hematocrit percentage (HCT%), (D) mean corpuscular volume (MCV), (E) red blood cell distribution width (RDWa), and (F) mean platelet volume (MPV). Data are presented as mean ± SD. Statistical analysis was performed using one‐way ANOVA followed by Dunnett's multiple comparisons test. *p < 0.05, **p < 0.01, ***p < 0.001, ****p < 0.0001 versus the non‐smoker control group.

Both cigarette and hookah smoking significantly increased RBC count (*p* = 0.005 and *p* < 0.001, respectively), hemoglobin (HGB; *p* < 0.001 for both), and hematocrit percentage (HCT%; *p* = 0.002 for cigarette and *p* < 0.001 for hookah) compared with controls (Figure 3A–C). Mean corpuscular volume (MCV) increased significantly in cigarette smokers only (*p* = 0.021), while hookah smokers showed no significant change (*p* > 0.05) (Figure 3D).

Red blood cell distribution width (RDWa) was significantly higher in hookah smokers (*p* < 0.001), but not in cigarette smokers (*p* = 0.253) (Figure 3E). Mean platelet volume (MPV) was also significantly elevated in hookah smokers (*p* = 0.003), but no significant change was observed in cigarette smokers (*p* > 0.05) (Figure 3F). Other hematological parameters including mean corpuscular hemoglobin (MCH), mean corpuscular hemoglobin concentration (MCHC), red cell distribution width (RDW%), platelet count (PLT), total white blood cell count (WBC), lymphocytes (LYM), mid‐sized cells representing monocytes and other less frequent leukocytes (MID), and granulocytes (GRA), reported as both absolute counts and percentages, did not show significant change (Table 1).

**TABLE 1:** Comparison of hematological parameters between cigarette smokers, hookah smokers, and non‐smokers (controls).

Blood parameter	Control (*n* = 29)	Cigarette (*n* = 29)	Hookah (*n* = 29)
MCH (pg) – Mean corpuscular hemoglobin	27.47 ± 1.89	28.03 ± 1.36	28.09 ± 2.04
MCHC (g/dL) – Mean corpuscular hemoglobin concentration	33.27 ± 0.91	32.83 ± 1.02	33.81 ± 0.84
RDW (%) – Red cell distribution width	11.83 ± 0.90	12.28 ± 1.23	11.81 ± 0.67
PLT (×10^3^/μL) – Platelet count	220.1 ± 43.66	210.2 ± 37.52	206.9 ± 51.29
PCT (%) – Plateletcrit	0.193 ± 0.054	0.180 ± 0.024	0.194 ± 0.047
PDW (fL) – Platelet distribution width	11.65 ± 1.00	11.74 ± 1.20	11.81 ± 0.67
WBC (×10^3^/μL) – White blood cells	7.25 ± 1.11	7.67 ± 1.54	7.58 ± 1.01
LYM (×10^3^/μL) – Lymphocytes	2.29 ± 0.48	2.74 ± 0.90	2.43 ± 0.92
LYM (%) – Lymphocyte percentage	31.76 ± 4.92	32.92 ± 7.48	31.93 ± 8.11
MID (×10^3^/μL) – Monocytes and mid‐sized cells	0.445 ± 0.106	0.498 ± 0.153	0.452 ± 0.098
MID (%) – Mid‐sized leukocyte percentage	5.94 ± 1.36	6.19 ± 1.56	5.70 ± 0.70
GRA (×10^3^/μL) – Granulocytes	4.44 ± 0.84	4.47 ± 1.16	4.68 ± 0.64
GRA (%) – Granulocyte percentage	62.04 ± 5.29	60.26 ± 7.51	62.37 ± 8.33

*Note:* Data are presented as mean ± standard deviation (SD). Statistical analysis was performed using one‐way ANOVA followed by Dunnett's multiple comparisons test. No significant differences were observed in the following parameters.

### 3.3 Correlation Between Oral Microbiota and Hematological Parameters

Exploratory correlation analysis demonstrated strong positive associations between oral bacterial load and erythrocyte‐related parameters. Specifically, bacterial load showed strong correlations with RBC (*r* = 0.91), hemoglobin (HGB; *r* = 0.94), and hematocrit (HCT; *r* = 0.92), indicating that increased microbial burden coincides with elevated erythropoietic indices among smokers.

In contrast, weak correlations were observed between bacterial load and inflammatory or platelet‐related parameters, including WBC (*r* = 0.21), RDW (*r* = 0.34), and MPV (*r* = 0.29). These findings suggest that oral microbial alterations are more closely aligned with erythrocyte changes than with systemic inflammatory responses (Table 2).

**TABLE 2:** Exploratory correlation analysis between oral bacterial load (Log_10_ CFU/mL) and hematological parameters across study groups.

Parameter	Pearson correlation (*r*)	Strength of association	Interpretation
Red Blood Cell (×10^6^/μL)	0.91	Strong positive	Higher bacterial load associated with increased erythrocyte count
Hemoglobin (HGB, g/dL)	0.94	Strong positive	Strong association with erythropoietic response
Hematocrit (HCT, %)	0.92	Strong positive	Reflects increased oxygen‐carrying capacity
White Blood Cells (×10^3^/μL)	0.21	Weak positive	Minimal association with inflammatory response
Red cell Distribution Width (%)	0.34	Weak positive	Slight variation in red cell distribution
Mean Platelet Volume (fL)	0.29	Weak positive	Limited association with platelet activity

*Note:* Pearson correlation coefficients (*r*) were calculated using group‐level mean values. Strong positive associations were observed between bacterial load and erythrocyte‐related parameters (RBC, HGB, HCT), while weak correlations were observed with inflammatory and platelet‐related indices (WBC, RDW, MPV). Due to the use of group‐level data, these findings should be interpreted as indicative trends rather than definitive statistical relationships.

## 4 Discussion

The present study demonstrates that both cigarette and hookah smoking are associated with measurable alterations in oral microbial composition and selected hematological parameters among young adult males. These findings suggest that tobacco exposure may influence both local microbial ecology in the oral cavity and systemic physiological responses.

The oral microbiota consists of complex microbial communities residing in distinct microenvironments regulated by host and environmental factors. These communities maintain homeostasis; however, disruption leads to dysbiosis and disease development. The oral cavity can act as a reservoir for opportunistic pathogens implicated in systemic diseases such as inflammatory bowel disease (IBD), arthritis, colorectal and pancreatic cancers, and Alzheimer's disease [8].

In the present study, smokers exhibited significantly higher oral microbial load and altered microbial distribution compared with non‐smokers. Cigarette smokers showed the highest bacterial burden, followed by hookah smokers. This supports previous findings that smoking reshapes oral microbial abundance and interactions. Co‐occurrence analyses have shown enrichment of pathogenic microorganisms and reduction of beneficial species in smokers [10]. In Middle Eastern populations, heavy smoking is associated with functional and chemical shifts in oral microbiota that predispose individuals to respiratory diseases and increased relapse risk [26].

Smoking‐associated dysbiosis is characterized by enrichment of pathogenic anaerobic species such as *Fusobacterium*, *Prevotella*, *Porphyromonas*, and *Tannerella*, alongside depletion of non‐pathogenic bacteria like *Streptococcus sanguinis* [9, 11]. Although the present study used culture‐based methods, the observed increase in *Staphylococcus aureus* and *Candida albicans* in smokers aligns with the concept of pathogen enrichment under smoking‐induced stress conditions. Furthermore, cigarette smoke exposure has been linked to increased severity of bacterial infections, including pneumonia [27].

Hookah smoking also demonstrated significant effects on oral microbiota. Although the microbial load was lower than in cigarette smokers, notable alterations were still observed. Previous studies indicate that waterpipe smoking disrupts oral microbial diversity and biochemical balance, increasing susceptibility to infections, including *Helicobacter pylori* colonization [12, 13].

Hematological findings further support systemic effects of smoking. Both cigarette and hookah smoking significantly increased RBC count, hemoglobin, and hematocrit levels, consistent with previous reports [18, 19, 20]. These changes are commonly attributed to carbon monoxide exposure, which reduces oxygen‐carrying capacity and triggers compensatory erythropoiesis [14, 17]. Such adaptations increase the risk of polycythemia and cardiovascular complications [6, 7, 15].

Mean corpuscular volume (MCV) was significantly elevated in cigarette smokers but not in hookah smokers, aligning with prior studies [19, 28]. In contrast, Ahmed et al. [20] reported lower MCV values among smokers compared with non‐smokers, highlighting population‐level variability in smoking‐associated microcytic changes. Mean corpuscular hemoglobin (MCH) and mean corpuscular hemoglobin concentration (MCHC) did not differ significantly between groups in our analysis, consistent with Malenica et al. [19]. However, Ahmed et al. [20] documented significant increases in both MCH and MCHC among smokers, further underscoring discrepancies that may reflect differences in population characteristics, smoking patterns, or exposure intensity.

Platelet indices indicated that mean platelet volume (MPV) was significantly elevated in hookah smokers, aligning with findings by Kutlu and Demirbaş [29]. No significant MPV changes were detected among cigarette smokers in our cohort. Although some studies have reported increased MPV in cigarette users [30, 31], Ahmed et al. [20] documented lower MPV levels in smokers compared with non‐smokers. This discrepancy underscores variability in platelet responses across smoking patterns and study populations. Elevated MPV remains clinically important due to its well‐established association with heightened atherosclerotic risk.

In contrast, white blood cell (WBC) counts and differential leukocyte profiles did not significantly differ among smokers in our study, despite prior reports of elevated WBCs, monocytes, and granulocytes [19, 32, 33]. Ahmed et al. [20] similarly reported significantly higher WBC and neutrophil counts in smokers, which diverges from our findings and may reflect differences in smoking intensity, demographic characteristics, or underlying health status.

The observed alterations in oral microbiota and hematological parameters may reflect broader biological effects of tobacco exposure on host physiology and microbial ecosystems. Tobacco‐related toxicants can modify the oral microenvironment by altering redox balance and inflammatory responses, conditions known to influence microbial community structure and facilitate colonization by opportunistic organisms [3, 4]. Such environmental shifts may contribute to the microbial patterns detected in this study and are consistent with previous reports describing smoking‐associated dysbiosis of the oral microbiota [8, 10]. In addition, chronic exposure to combustion products such as carbon monoxide may affect oxygen transport and stimulate compensatory hematological responses, which could explain the changes observed in several blood indices. Although the present study was not designed to investigate these pathways directly, the findings are compatible with mechanisms proposed in earlier studies. Future investigations combining molecular approaches and comprehensive microbiome profiling would help clarify the biological processes underlying these smoking‐associated alterations.

Exploratory correlation analysis revealed strong positive associations between oral bacterial load and erythrocyte‐related parameters (RBC, HGB, HCT), while weak correlations were observed with inflammatory and platelet indices. These findings suggest that oral microbial alterations are more closely aligned with erythrocyte changes than systemic inflammatory responses. However, these correlations should be interpreted cautiously.

Although smoking induces systemic biological effects, current evidence does not support a direct mechanistic link between oral microbiota changes and erythropoiesis. Tobacco exposure, particularly through hookah smoking, generates high levels of toxicants including carbon monoxide and oxidants that induce oxidative stress and inflammation [5]. Notably, waterpipe smoking may produce higher levels of these toxicants than cigarette smoking, amplifying systemic effects.

Biomarker‐based research further indicates that the physiological effects of smoking are best assessed through direct biochemical indicators such as carbon monoxide and nicotine metabolites [34]. Therefore, the hematological changes observed in this study are more plausibly explained by systemic exposure to smoking‐related toxicants rather than oral microbial alterations.

Accordingly, the correlations identified in this study are more plausibly explained by shared exposure to smoking‐related toxicants, which may simultaneously influence both oral microbial composition and hematological parameters, rather than a direct causal relationship between oral dysbiosis and erythrocytosis. This interpretation is further supported by the weak correlations observed with inflammatory markers (WBC, RDW, MPV), indicating that these associations likely reflect parallel biological responses rather than a unified mechanistic pathway. Therefore, these findings should be considered exploratory and interpreted cautiously, and future studies incorporating direct biomarkers (e.g., carboxyhemoglobin) and molecular microbiome analyses are required to clarify the underlying mechanisms.

Overall, the combined alterations in oral microbiota and hematological parameters highlight the dual local and systemic impact of smoking. These changes may contribute to increased susceptibility to infections, cardiovascular disease, and other chronic conditions.

In conclusion, both cigarette and hookah smoking significantly alter oral microbial composition and hematological parameters, with distinct patterns observed between smoking types. Cigarette smoking was associated with higher microbial load and erythrocyte indices, while hookah smoking showed notable effects on platelet‐related parameters. These findings emphasize that hookah smoking is not a safer alternative to cigarette smoking. The combined microbial and hematological alterations underscore the need for targeted public health interventions and awareness programs, particularly in regions where both smoking practices are prevalent. Future longitudinal and mechanistic studies are required to better understand causality and underlying biological pathways.

This study is limited by its cross‐sectional, single‐center, male‐only design, which restricts generalizability. Culture‐based methods captured only the cultivable microbiota, and molecular analyses (e.g., 16S rRNA sequencing) were not performed. Mechanistic biomarkers such as carboxyhemoglobin and erythropoietin were not measured, and the correlation analysis was exploratory and based on group‐level data.

## Author Contributions

All authors read and approved the final manuscript.

## Funding

No specific external funding was received for this work.

## Ethics Statement

Ethical approval for this study was obtained from the Ethics Committee of Cihan University Sulaimaniya, Iraq (Reference No. HS‐1‐2018). All procedures involving human participants were conducted in accordance with the ethical standards of the national research committee and with the 1964 Declaration of Helsinki.

## Consent

Written informed consent was obtained from all participants prior to sample collection.

## Conflicts of Interest

The authors declare no conflicts of interest.

## Data Availability

The datasets generated and/or analyzed during the current study are available from the corresponding author upon reasonable request.
